# Effect of Rh Doping on Optical Absorption and Oxygen Evolution Reaction Activity on BaTiO_3_ (001) Surfaces

**DOI:** 10.3390/molecules29112707

**Published:** 2024-06-06

**Authors:** Talgat M. Inerbaev, Aisulu U. Abuova, Zhadyra Ye. Zakiyeva, Fatima U. Abuova, Yuri A. Mastrikov, Maksim Sokolov, Denis Gryaznov, Eugene A. Kotomin

**Affiliations:** 1 Department of Technical Physics, L.N. Gumilyov Eurasian National University, Astana 010000, Kazakhstan; talgat.inerbaev@gmail.com (T.M.I.); jadira.zakieva@mail.ru (Z.Y.Z.); abuova_fu@enu.kz (F.U.A.); 2 Vernadsky Institute of Geochemistry and Analytical Chemistry, Russian Academy of Science, 119991 Moscow, Russia; 3 Institute of Solid State Physics, University of Latvia, LV-1063 Riga, Latvia; yuri@umd.edu (Y.A.M.); makcsokolov@gmail.com (M.S.); kotomin@latnet.lv (E.A.K.)

**Keywords:** electrocatalysis, photocatalysis, energy storage and conversion, electrode materials, water splitting

## Abstract

In the present work, we investigate the potential of modified barium titanate (BaTiO_3_), an inexpensive perovskite oxide derived from earth-abundant precursors, for developing efficient water oxidation electrocatalysts using first-principles calculations. Based on our calculations, Rh doping is a way of making BaTiO_3_ absorb more light and have less overpotential needed for water to oxidize. It has been shown that a TiO_2_-terminated BaTiO_3_ (001) surface is more promising from the point of view of its use as a catalyst. Rh doping expands the spectrum of absorbed light to the entire visible range. The aqueous environment significantly affects the ability of Rh-doped BaTiO_3_ to absorb solar radiation. After Ti→Rh replacement, the doping ion can take over part of the electron density from neighboring oxygen ions. As a result, during the water oxidation reaction, rhodium ions can be in an intermediate oxidation state between 3+ and 4+. This affects the adsorption energy of reaction intermediates on the catalyst’s surface, reducing the overpotential value.

## 1. Introduction

The growing demand for environmentally friendly and cost-effective energy sources has led to intensive research into various renewable energy sources. In this regard, photoelectrochemical hydrogen generation through water splitting has emerged as a promising avenue due to its affordability and environmental friendliness. In 1972, Honda and Fujishima first reported hydrogen production through photochemical water splitting using the semiconductor TiO_2_ [1]. Since then, this phenomenon has been extensively studied, and numerous materials and water-splitting systems have been developed. In the process of photoelectrochemical (PEC) water splitting, hydrogen is produced from water by using sunlight and specialized semiconductors called PEC materials. These materials directly split water molecules into hydrogen and oxygen using light energy.

An integrated PEC system consists of light absorbers, electrocatalysts for the hydrogen evolution reaction and the oxygen evolution reaction (OER), electrolytes, and membranes. This system can be used to efficiently produce hydrogen fuel from sunlight, especially through the photo-electrolysis of water, generating sustainable hydrogen and oxygen. However, the key to achieving viable PEC solar water splitting lies in carefully selecting semiconductive electrode materials. These materials must have low band gaps and exceptional stability and be inexpensive. This strategic choice allows for the absorption of a greater amount of visible light, thereby enhancing the overall efficiency of the process.

Perovskite-based materials are widely regarded as highly efficient photocatalysts for water splitting due to their adjustable electronic properties [2,3,4,5,6]. In addition, perovskite materials comprise environmentally benign and inexpensive elements abundant on Earth [7,8]. Recently, there has been increased focus on using perovskites as cost-effective catalysts for water electrolysis due to a deeper comprehension of the rapport between electronic structure and reactivity [9,10]. As a new category of perovskite derivatives, layered Ruddlesden–Popper perovskites are currently attracting growing research attention [11,12].

BaTiO_3_, utilized as a crystal in non-linear optics, dielectric ceramics, and piezoelectric materials, is among the ferroelectric oxides that have been the subject of extended scientific inquiry [13]. The optical band gap of pristine BaTiO_3_ is 3.2–3.4 eV, much larger than the activation energy of 1.23 eV required for water splitting [14]. Therefore, the use of bare titania for solar energy harvesting is not efficient. Band gap excitation requires ultraviolet irradiation (UV); however, UV light accounts for only 4% of the solar spectrum compared to the 45% that is visible. So, any shift in optical response to the visible range will have a profound positive effect on the photocatalytic efficiencies of BaTiO_3_ materials.

There have been reports of water electrolysis using BaTiO_3_ electrodes [15,16]. Ni-supported BaTiO_3_ exhibits activity for CO_2_ reformation [17], Pd-modified BaTiO_3_ efficiently catalyzes NO_x_ reduction [18], and Cr-modified BaTiO_3_ catalyzes the reduction of nitrobenzene and aniline [19]. Several methods are used for enhancing the electronic properties of barium titanate for electrocatalysts application. Catalyst performance could be, in principle, improved using different promoters like W, Mn, and Fe [20,21,22]. According to a theoretical study [23], Fe_Ti_ and Ni_Ti_ substitutions increased electrical conductivity and reduced overpotentials for the OER. Xie et al. [24] revealed experimentally that applying a 2% Mo doping to BaTiO_3_ results in a reduction in the optical bandgap that activates its photo-catalytic performance. Eu-doped BaTiO_3_ nanoparticles show remarkable electrochemical performance towards the oxygen evolution reaction (OER) and excellent stability over 2000 cyclic voltammetry cycles [25].

Rh doping is one of the most effective methods that enables one to produce a visible-light-responsive photocatalyst [26,27,28]. Related to BaTiO_3_, rhodium-doped SrTiO_3_ exhibits remarkable photocatalytic efficiency in the process of H_2_ evolution from an aqueous methanol solution under visible light irradiation, outperforming all other visible-light-activated oxide photocatalysts [27]. Bhat et al. [29] suggested that Rh-doped BaTiO_3_ resulted in the formation of mid-gap electronic states, causing a reduction in the band gap of BaTiO_3_ while simultaneously avoiding the formation of recombination centers. As seen from the studies mentioned above, the research on Rh-modified impacts on the catalyst properties of BaTiO_3_ is limited and requires more detailed consideration. In light of these novel findings, in the current article, we investigate the degree to which a minor modification can be made to the chemical composition of the surface of barium titanate (BaTiO_3_) to tune its catalytic reactivity. This study focuses on the optical absorption and catalytic performance towards OER of pure and Rh-modified tetragonal BaTiO_3_ structures.

## 2. Theoretical Surface and Thermodynamic Model

### 2.1. Structure Models

In this work, the tetragonal BaTiO_3_ phase, which is not energetically favorable at a temperature of zero but exists at room temperature, was used in the modeling conducted. Initial crystal structure was taken from the Materials Project database [30]. To make the (001) surface models of BaTiO_3_, slabs with eleven layers of TiO_2_ and BaO that are symmetric concerning the mirror plane were used. The end of one of these slabs had BaO planes for the crystal and was a supercell containing 108 atoms. The second slab terminated in TiO_2_ planes containing 112 atoms. The (001) surface was chosen because it is the most energetically favorable for both TiO_2_ and BaO terminations [31]. A vacuum layer measuring 15 Å thick was applied perpendicular to the slabs to avoid artificial interactions between the slab and its periodic images.

**Figure 1:**
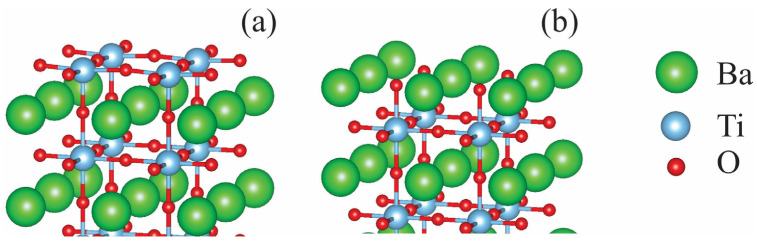
(**a**) TiO_2_- and (**b**) BaO-terminated (001) surfaces of tetragonal BaTiO_3_.

Even though these slabs are not stoichiometric, they maintain symmetry when the Ba/Ti atoms are substituted with Rh on the outermost layer, preventing the system from having a dipole moment. Due to periodic boundary conditions, this dipole moment may significantly distort the calculated energy values of the systems. These two slab ends (TiO_2_ and BaO) are the only possible terminations of (001) surfaces for the BaTiO_3_ perovskite lattice structure, as shown in Figure 1. Replacing the Ba atoms on a BaO-terminated surface results in the doping atom formally entering the Rh_Ba_^2+^ state. Experimentally, Rh^3+^ and Rh^4+^ ions have been detected when BaTiO_3_ is doped [32], so neutral OH groups were added to the surface to change Rh_Ba_^2+^ into Rh_Ba_^3+^. The present study focuses on the TiO_2_-terminated surface because it has recently been shown that the BaO-terminated surface is also unstable under operating conditions [23].

### 2.2. Thermodynamic Description

Under the standard conditions (T = 298 K, p = 1 bar, pH = 0), the equilibrium thermodynamic potential for water oxidation required to produce oxygen (H_2_O → 1/2O_2_ + 4H^+^ + 4e^−^) is 1.23 V vs RHE (the reference electrode is further omitted for brevity). In practice, a potential above 1.23 V is required for this reaction. For heterogeneous catalysts, this additional potential is referred to as the overpotential η. 

The catalytic oxygen evolution reaction (OER) via water oxidation is divided into four fundamental reaction steps, wherein each step entails the exchange of an electron–proton pair (where * denotes the adsorption site of the catalyst) [33,34]:

(1a) $$2H_{2}O+\;*\;⇌{\mathrm{OH}}^{*}+H_{2}O+H^{+}+e^{-}$$

(1b) $${\mathrm{OH}}^{*}+H_{2}O⇌O^{*}+H_{2}O+H^{+}+e^{-}$$

(1c) $$O^{*}+H_{2}O⇌{\mathrm{OOH}}^{*}+H^{+}+e^{-}$$

(1d) $${\mathrm{OOH}}^{*}⇌\;*+O_{2}+H^{+}+e^{-}$$

Using the normal (computational) hydrogen electrode approach, the reaction free energy ∆*G* of the charge transfer reaction H* ⇌ * + H^+^ + e^−^ under standard ambient conditions is equal to the ∆*G* of the H* ⇌ * + 1/2H_2_ reaction. The reactions’ Gibbs free energy for steps ∆G_1_, ∆G_2_, ∆G_3_, and ∆G_4_ in Equation (1) can be expressed as

(2) $$\Delta G_{1}=\Delta G_{\mathrm{OH}}-eU+\Delta G_{H+}\left(\mathrm{pH}\right)\;\Delta G_{2}=\Delta G_{O}-\Delta G_{\mathrm{OH}}-eU+\Delta G_{H+}\left(\mathrm{pH}\right)\;\Delta G_{3}=\Delta G_{\mathrm{OOH}}-\Delta G_{O}-eU+\Delta G_{H+}\left(\mathrm{pH}\right)\;\Delta G_{4}=4.92\left[\mathrm{eV}\right]-\Delta G_{\mathrm{OOH}}-eU+\Delta G_{H+}\left(\mathrm{pH}\right)$$

where *U* is the potential measured against a normal hydrogen electrode (NHE) under standard conditions. The free energy change of the protons relative to the NHE at non-zero pH is represented by the Nernst equation as Δ*G*_H+_(pH) = −*k_B_T* ln(10) × pH. The Gibbs free energy differences in Equation (2) include zero-point energy (ZPE) and enthropy corrections according to $\Delta G_{i}=\Delta E_{i}-T\Delta S_{i}+\Delta ZPE_{i}-eU$. Entropic contributions under standard conditions were taken from the CRC Handbook [35]. The Supporting Information for Ref. [34] also includes these values. Energy differences Δ*E*_i_ calculated relative to H_2_O and H_2_ (at *U* = 0 and pH = 0) are approximated as follows:

(3) $$\Delta E_{\mathrm{OH}}=E\left({\mathrm{OH}}^{*}\right)-E\left(*\right)-\left[E\left(H_{2}O\right)-\frac{1}{2}E\left(H_{2}\right)\right]\;\Delta E_{O}=E\left(O^{*}\right)-E\left(*\right)-\left[E\left(H_{2}O\right)-E\left(H_{2}\right)\right]\;\Delta E_{\mathrm{OOH}}=E\left({\mathrm{OOH}}^{*}\right)-E\left(*\right)-\left[2E\left(H_{2}O\right)-\frac{3}{2}E\left(H_{2}\right)\right]$$

The theoretical overpotential can then be readily defined as

(4) $$\eta =max\left[\Delta G_{i}\right]/e-1.23\;\left(V\right)$$

The overpotential represented by Equation (4) is simply a thermodynamic quantity. Due to the lack of activation barriers, experimentally determined overpotential values cannot be directly compared with theoretical ones. In addition, experiments are usually carried out using electrodes containing nanoparticles of the used material, whose active surface’s exact value is difficult to determine.

## 3. Results and Discussion

### 3.1. Effect of Doping on Ground-State Electronic Properties

*Geometry modification.* The computed lattice parameters for the bulk tetragonal BaTiO_3_ are a_0_ = 4.0381 Å and c_0_ = 4.0999 Å. Several of the experimental data that are accessible are comparable to our findings: a_0_ falls within the range of 3.9860 Å to 3.9905 Å, and c_0_ spans from 4.0170 Å to 4.0412 Å [36,37,38,39,40].

**Figure 2:**
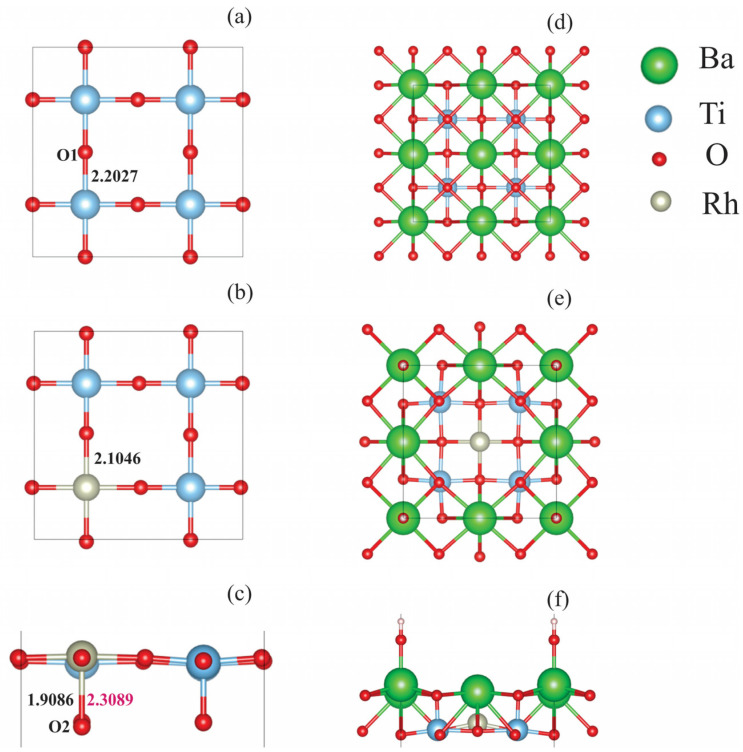
(**Left**): Top view of the outermost layer of TiO_2_-terminated (**a**) undoped and (**b**) Rh-doped surfaces. The numbers indicate the distance (Å) between the (**a**) Ti and (**b**) Rh atoms and the nearest surface oxygen atoms (O1). (**c**) Side view of a doped TiO_2_-terminated surface (Ba ions omitted); the numbers indicate the interatomic distance between the metal atoms (Ti: black, Rh: pink) and subsurface oxygen (O2). (**Right**): Top view of the two upper layers of BaO-terminated (**d**) undoped and (**e**) Rh-doped surfaces. Side view of a doped BaO-terminated surface (**f**).

The TiO_2_-terminanted surface replacement of Ti^4+^ with Rh^4+^ leads to slight distortion of the lattice, as shown in Figure 2a–c. Each surface Ti^4+^ ion is surrounded by four neighboring surface oxygen ions (O1) and one nearest-subsurface oxygen (O2). All Ti^4+^-O1 distances are the same and are 2.2027 Å, while the Ti^4+^-O2 bond lengths are 1.9086 Å. After the Ti^4+^→Rh^4+^ substitution, the Rh_Ti_^4+^-O1 bond lengths are 2.1046 Å, and the Rh_Ti_^4+^-O2 distance is 2.3089 Å. Substitution energy, Ti^4+^→Rh^4+^, is calculated as follows:

*E*_def_ = (*E*(Rh-doped) + *E*(Ti) − *E*(undoped) − *E*(Rh))/2,

where *E*(undoped) and *E*(Rh-doped) are the calculated energies of the pristine and doped slabs, and *E*(Ti) and *E*(Rh) are the energies per atom for metals *hcp*-Ti and *bcc*-Rh. The calculations yield the value Edef = 7.212 eV per Rh atom. This value is typical for this type of substitution. Thus, the previously calculated value of the Ti^4+^→Ru^4+^ substitution energy is 6.424 eV per Ru atom [41].

In the case of Ba^2+^ → Rh^3+^ + OH^−^ substitution, a much stronger distortion of the surface structure occurs. After geometry optimization, Rh_Ba_^3+^ ions are displaced, moving from the surface layer deep in the slab to the subsurface layer, forming bonds with oxygen ions in this layer (Figure 2d–f). In this case, in the next atomic layer under the Rh^3+^ ion, Ba^2+^ is present. This finding shows that even if there were a BaO-terminated surface, the doping ion Rh_Ba_^3+^ would not be on the surface layer. This would make this site less likely to be able to catalyze water-splitting reactions.

**Figure 3:**
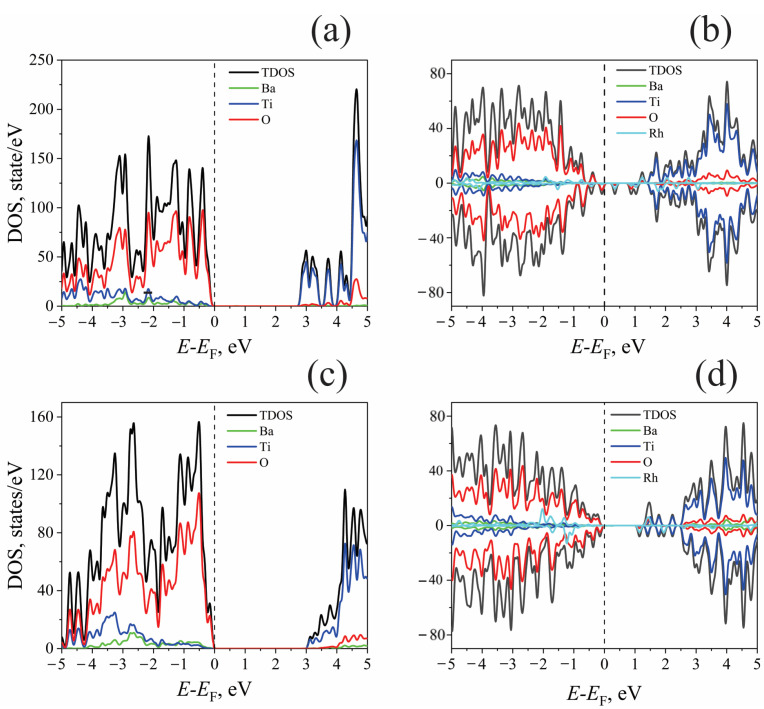
Total and partial densities of states for bare and doped TiO_2_- and BaO-terminated surfaces. Top: (**a**) undoped TiO_2_-terminated surface; (**b**) Rh-doped TiO_2_-terminated surface. The contribution of the surface nearest to the oxygen atoms of Rh, O(Rh), is highlighted. Bottom: (**c**) undoped BaO-terminated surface; (**d**) Rh-doped BaO-terminated surface. *E*_F_: Fermi energy.

*Electronic density of states*. The HSE06-calculated electronic structures of the doped and undoped models are schematically summarized in Figure 3. Figure 3 presents the total (TDOS) and partial densities of states of the bare and doped TiO_2_- and BaO-terminated surfaces. For both bare surfaces, the O-2p states predominately form a valence band, whereas the Ti-4d states form the conduction band minimum. The O-2p→Ti-3d transitions thus determine optical absorption for undoped BaTiO_3_. The calculated band gaps for the undoped models are 2.8 eV and 3.0 eV for the TiO_2_- and BaO-terminated surfaces, respectively. The different stoichiometries of the studied models account for this variation in the calculated bandgap values. However, the bandgap values obtained from the DOS calculations do not coincide with the results of the optical spectra calculations, which will be shown below when analyzing the optical absorption spectra.

Doping the TiO_2_-terminated surface results in additional levels due to the Rh-4*d* states appearing in the band gap (Figure 3b). The Rh^4+^ ion also changes the electronic states of the oxygen atoms that are closest to it. This causes the O-2*p* peaks to appear in the calculated DOS near the valence band maximum. This effect also results in an additional reduction in the band gap. When doping a BaO-terminated surface, in addition to the Rh-4*d* states in the bandgap, the Ti-3*d* states appear near the minimum of the conduction band; titanium ions close to the Rh ion in the subsurface layer give rise to these states. As Rh^4+^ shifts from the surface to the layer below, it breaks the bonds between the dopant and the surface oxygen ions. This creates more O-2*p*-induced peaks in the DOS near the top of the valence band (Figure 3d).

### 3.2. Optical Absorption

**Figure 4:**
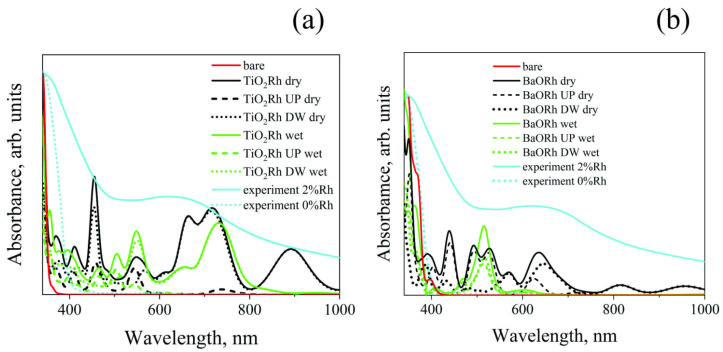
Optical absorption of undoped and Rh-doped (**a**) TiO_2_- and (**b**) BaO-terminated surfaces. Black and blue lines correspond to dry and wet surfaces, respectively. The solid lines illustrate total optical absorption, while dashed and dotted lines correspond to the contributions of spin-up (UP) and spin-down (DW) electronic states. Orange lines refer to experimental data adapted from Ref. [32].

The effect of doping on optical absorption is shown in Figure 4. Both dry and wet surfaces are considered. The presence of Rh^4+^ ions (Figure 4a) on the TiO_2_-terminated surface substantially changes the optical absorption spectrum due to the DOS changes discussed above. Although the DOS calculations for an undoped surface yield a band gap of 2.8 eV, the optical absorption threshold is 3.35 eV (370 nm). This difference exists because for optical transitions of the O-2*p*→Ti-3*d* type in the energy range of 2.8–3.1 eV, the oscillator strengths calculated using Equation (8) are equal to zero or assume negligibly small values. As a result, the optical absorption threshold value for the undoped structure is in good agreement with experimental data [32]. The spin-down O-2*p→*Rh-4*d* transitions on a Rh-doped surface absorb light in the long-wavelength range. In the short-wavelength range, optical absorption occurs due to the O-2*p*→Ti-3*d* transitions. The optical absorption peak at 900 nm is suppressed in the aqueous environment and the absorption at 450 nm is significantly reduced. In this case, optical absorption increases in the 500–550 nm range. A comparison of the calculated data and the experimental results obtained after the 2 mol% doping of BaTiO_3_ is presented [42]. The agreement between the theoretical and experimental results can be considered good since modeling shows that in the case of replacing the surface Ti^4+^ ion with Rh_Ti_^4+^, optical absorption occurs in a wide range of frequencies of electromagnetic radiation. Up to this stage, our model does not consider the role that Rh^4+^ ions inside the slab might play in optical absorption. In this case, these ions would not be on the sample’s surface, and aqueous media would not affect their electronic states. Below, we present an analysis and its results for the situation when the Rh ions reside inside the slab.

The optical absorption threshold value for the BaO-terminated surface is the same as that found by directly estimating the bandgap value from the DOS calculation and amounts to 415 nm (2.99 eV). The optical absorption at longer wavelengths is also due to the O-2*p*→Rh-4*d* and O-2*p*→Ti-3*d* transitions. In this case, in contrast to the TiO_2_-terminated surface, in the 400–520 nm wavelength range (2.4–3.1 eV), there is a contribution from the Rh-4*d*→Ti-3*d* transitions. This finding agrees with experimental data [32]. The transitions discussed here suggest that the electronic transitions from the Rh^3+^ ions to the conduction band are possible, even though Rh^4+^ usually plays the role of a trapping center [43]. 

Since the BaO-terminated surface was probably unstable but we knew that the Rh^3+^ ions help with optical absorption, we also looked at a model where the Rh ions were put inside the BaO-terminated slab instead of the slab surface. To ensure the Rh^3+^ oxidation state was obtained, neutral OH groups were added to the surface.

**Figure 5:**
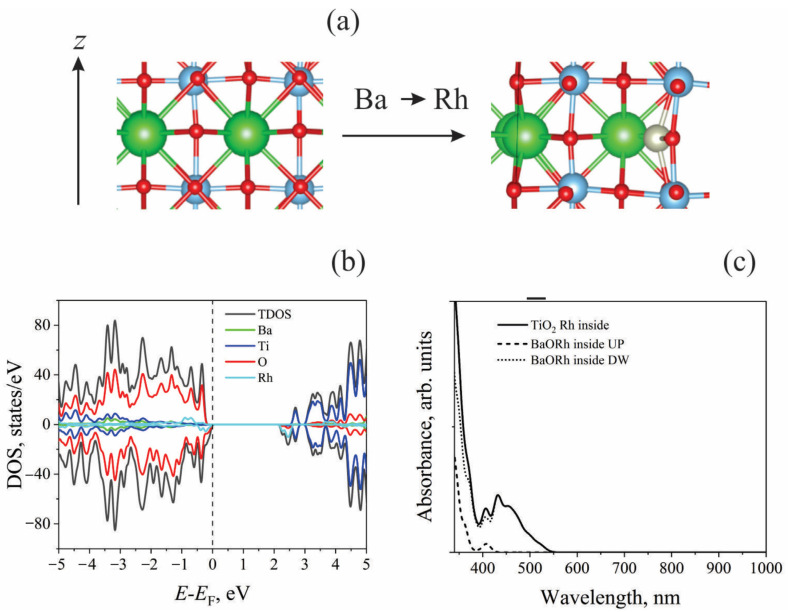
(**a**) Change in the arrangement of ions in the slab after Ba was replaced with Rh; (**b**) electronic DOS for relaxed slab; (**c**) optical absorption spectrum for the model investigated. Dashed and dotted lines represent optical absorption by spin-up and spin-down states. The solid line illustrates total absorption.

The results of geometry optimization, DOS, and optical absorption calculations are presented in Figure 5. Figure 5a shows how the atomic structure changes when rhodium is added after the structure’s geometry has been optimized. As in the case of the BaO-terminated surface (Figure 2e,f), the Rh_Ba_^3+^ ion is shifted towards the TiO_2_ plane. Unlike in the previous case, the displacement occurs in a direction parallel to the surface plane since the lattice parameters in this direction are lower than those perpendicular to the direction. The DOS analysis (Figure 5b) shows that the nature of the bottom of the conduction band is due to the Rh-4*d* and Ti-3*d* levels. The Ti-3*d* states are localized on Ti atoms located near Rh. So, optical absorption (Figure 5c) begins at 550 nm (2.25 eV) and is caused by transitions from O-2*p* to Rh-4d for the spin-down states. Furthermore, at wavelengths of approximately 500 nm (2.5 eV) and shorter, Rh-4*d*→Ti-3*d* transitions are possible. Thus, the experimentally observed Rh-4*d*→Ti-3*d* transitions [32] are most likely caused by Rh^3+^ ions inside the sample.

### 3.3. OER over Pristine and Rh-Modified BaTiO_3_

The above results indicate that Rh doping dramatically improves the ability of BaTiO_3_ to absorb sunlight in the visible range. The TiO_2_-terminated surface is also more stable regarding the Ti^4+^→Rh^4+^ change, while the Ba-terminated surface’s Rh^3+^ ion position is less stable. It was previously shown that the TiO_2_-terminated surface is stable under operating conditions. In contrast, the BaO-terminated surface is unstable concerning Ba dissolution at a wide range of pH values and potentials [32]. Based on these results, we evaluated the reaction-free energy profile for the OER on the TiO_2_-terminated surface of BaTiO_3_, as described in the Models Section 2.

**Figure 6:**
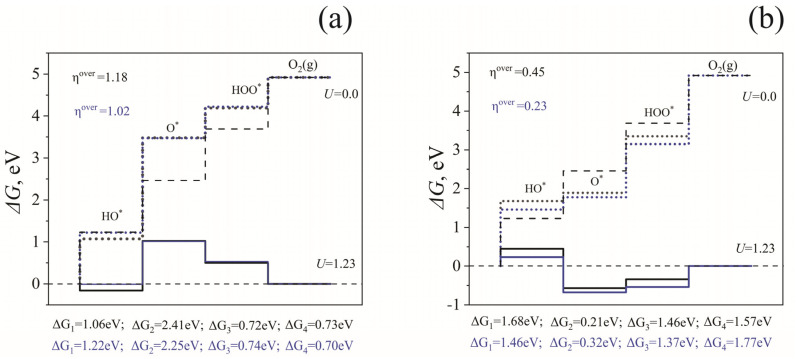
Standard free energy diagram for the OER at zero potential (U = 0, dotted lines) and equilibrium potential for oxygen evolution (*U* = 1.23 V, solid lines) at pH = 0 and T = 298 K. Black and blue lines show data for dry and wet surfaces, respectively. Dashed lines correspond to the ideal catalyst.

Figure 6 displays the free energies of water oxidation reactions on a pure and Rh-modified TiO_2_-terminated BaTiO_3_ surface at zero potential and equilibrium potential of 1.23 V vs RHE. (Equation (1)). The oxidation reaction of a single water molecule is considered both on a dry surface and considering the influence of the aqueous environment. On a bare TiO_2_-terminated surface, an overpotential of 1.18 V was found when the surface was dry. This value is close to the earlier-reported one calculated on the same surface, equal to 1.22 V [23]. Due to the aqueous environment, this value reduced to 1.08 V. For the Rh-modified surface, the overpotential values were 0.45 and 0.23 V for dry and wet surfaces, respectively, which implies that Rh doping improves catalytic activity. The obtained values are close to those for NiO_x_ films, in which cerium was used as a dopant and gold was employed as a metal support [44].

Since the efficiency of the photocatalyst in the process of the water oxidation reaction is determined by the energies of the interaction of intermediate reaction products with the surface (Equation (1)), it is necessary to analyze the oxidation states of active sites during the water-splitting process. The results regarding the Bader analysis and the spin states of active sites on the surface of the catalyst and the intermediate reaction products are given in Table 1. The number of active sites on the surface also includes the nearest neighboring ions, O1 and O2, since their charges and spin states change on the doped surface during the reactions represented by Equation (1). 

In the case of an unmodified TiO_2_-terminated surface, the charge and spin states of the catalyst ions change slightly during the oxidation of water, both in the case of dry and wet surfaces. The active site of a titanium ion is always in the 4+ oxidation state, and its nearest neighbors are in the O^2−^ state. An aqueous environment noticeably affects only the intermediate reaction product O*, reflected in a decrease in overpotential at this reaction step. In a sense, the electronic Ti^4+^ ion is too rigid in terms of its properties and cannot adjust its electronic structure to optimize the water-splitting process. Surface modification with Rh solves this problem.

When replacing the surface titanium ion with rhodium, the dopant also affects its nearest neighboring O1 and O2 ions. The data in Table 1 show that as the absolute value of the Bader charge on the O1 and O2 ions decreases, a non-zero magnetic moment also appears on these ions. This indicates a charge transfer from the O1 and O2 ions to the dopant. The spin state of the Rh ion also shows that it is not in the 4+ oxidation state since in this latter case its formal magnetic moment is 1 μ_B_ in the low-spin state (4d^5^). The present calculations suggested a value of 1.59 μ_B_ for the spin magnetic moment of Rh, which means Rh is in the 3+ oxidation state; i.e., the formal magnetic moment is 2 μ_B_ in the intermediate spin state. This deviation from the formal value is associated with the charge transfer from O1 and O2 to the doping cation. During the oxidation of water, the magnetic moment of Rh is 1.04 (O*) and decreases to 0.85 (OH*) and 0.75 (OOH*) μ_B_. This can be interpreted as the oxidation state of Rh undergoing a change from 3+ (O*) to 4+ (OH* and OOH*).

Because of the water oxidation reaction, the oxidation state and spin magnetic moment of the ions on the catalyst surface change, and the reaction intermediates change with them. The ability of Rh and the surrounding ions on the surface to change their electronic properties leads to more efficient water oxidation. The influence of the aqueous environment significantly affects the behavior of OH* species, which, in turn, leads to a decrease in the overpotential.

**Figure 7:**
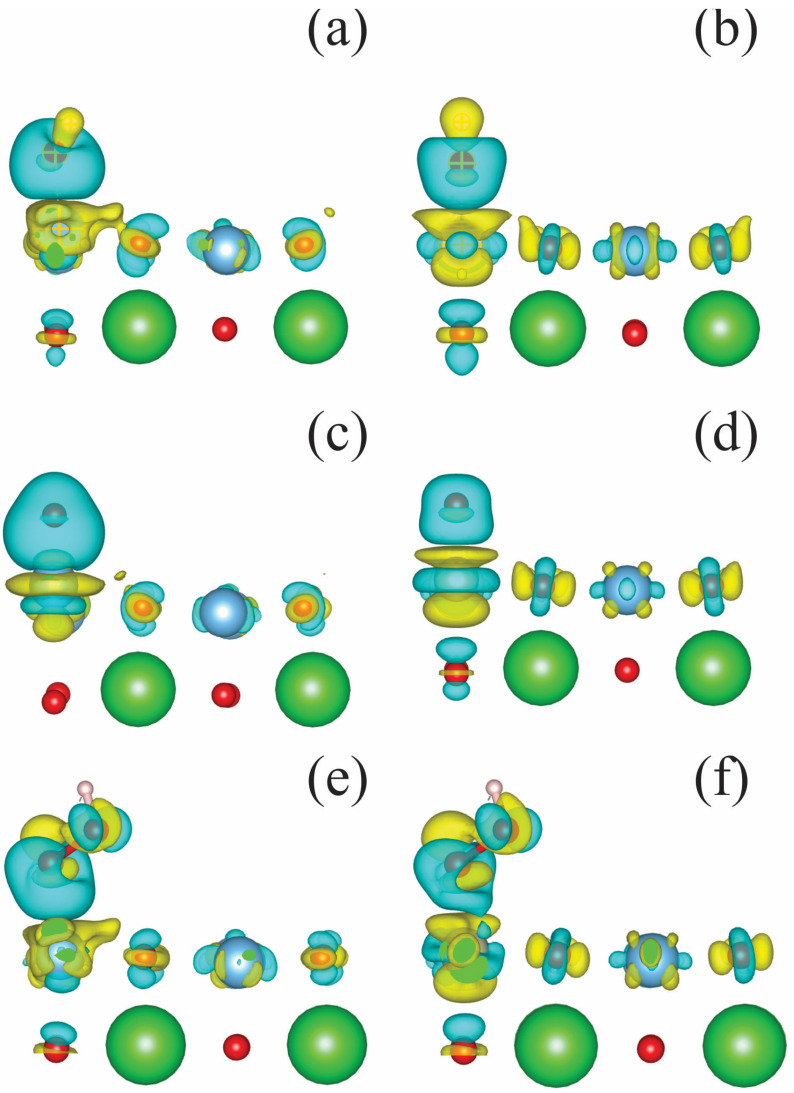
Equation (5) calculates the charge transfer between the TiO_2_-termiated catalyst surface and the intermediate reaction products. A side view of the surface of the top two layers is presented. OH adsorbed on (**a**) undoped and (**b**) Rh-doped surfaces; O adsorbed on (**c**) undoped and (**d**) Rh-doped surfaces; and HOO adsorbed on (**e**) undoped and (**f**) Rh-doped surfaces. The yellow and blue clouds indicate the isocontours of positive and negative values of the electron charge density, respectively.

Figure 7 shows how the electronic charge density redistributes between the dry TiO2-terminated surface and the reaction intermediates. The charge transfer ∆Q can be calculated using the formula given below:

∆*Q* = *Q*_SA_ − *Q*_S_ − *Q*_A_, (5)

Here, *Q*_SA_, *Q*_S_, and *Q*_A_ represent the spatial charge density distributions for systems wherein the intermediate reaction products are adsorbed on the surface of the catalyst, the bare catalyst surface, and the adsorbed species treated separately from the catalyst, respectively. The oxygen atoms of the adsorbed species are mainly responsible for the charge transfer. These findings are summarized in Table 1.

We compared the geometry of optimized undoped and doped TiO2-terminated catalyst surfaces with adsorbed reaction intermediates. Table 2 summarizes the distances between the adsorbents and the surface. In all cases, doping decreases the distance between the adsorbent and the catalyst surface, except for OOH species adsorption. There is a significant difference between the undoped and doped surfaces in regard to the orientation of the adsorbed OH group. In the case of adsorption on an undoped surface, the angle $\hat{\mathrm{TiOH}}$ = 128°, while in the case of a doped surface, the OH group is directed perpendicular to the surface, and $\hat{\mathrm{RhOH}}$ = 180°.

To illustrate the effect of an aqueous environment, we calculated the spatial distribution of charge density difference between wet and dry TiO_2_-terminated surfaces; this distribution was calculated as follows:

(6) $$\Delta Q=Q_{wet}-Q_{dry}$$

**Figure 8:**
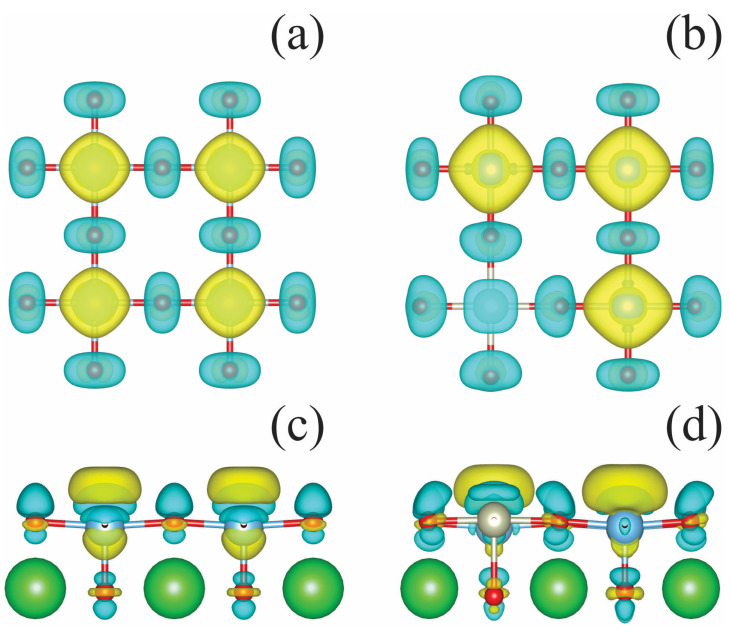
Equation (6) calculates the charge transfer between wet and dry TiO_2_-termiated catalyst surfaces. Top view of the upper layer of the (**a**) undoped and (**b**) Rh-doped surfaces; side view of the two upper layers of the (**c**) undoped and (**d**) Rh-doped surfaces. The yellow and blue clouds indicate the isocontours of positive and negative values of the electron charge density, respectively.

Figure 8 illustrates the obtained results. The aqueous environment leads to a transfer of electron density from surface oxygen ions to titanium ions. In an aqueous environment, the doped structure experiences a decrease in the electron density on Rh.

Although the predicted overpotential values are small, in practice, implementing an electrode with such indicators will take much work. Here, we consider the ideal case of doping wherein all Rh ions are located on the surface of BaTiO_3_ at 1.8 at.% doping. In practice, a significant portion of the doping atoms will occupy sites inside the nanoparticles. When the degree of doping is raised to increase the concentration of surface Rh ions, the hexagonal BaTiO_3_ phase forms [32]. The catalytic properties of the hexagonal phase still need to be studied.

Optimizing catalyst activity hinges on the discovery of a catalyst with a significantly larger surface area and a higher concentration of dopant atoms. These things are very important for making the tetragonal phase of BaTiO_3_ doped with Rh function as efficiently as possible. Ref. [32] serves as a valuable guide for determining the optimal doping level. The results show that adding 8 mol% Rh changes 85% of the tetragonal BaTiO_3_ phase into a hexagonal structure. When synthesizing BaTiO_3_ crystals with a Rh content below the specified level, grinding the crystals becomes necessary. This process enhances the working surface area of the catalyst, thereby increasing the likelihood of detecting Rh atoms on the surfaces of the resulting nanoparticles. By following this procedure, we can secure the most efficient catalyst based on BaTiO_3_ doped with Rh.

## 4. Computational Details

All the calculations were performed with the ab initio plane wave computer code VASP [45,46] using the projector-augmented plane wave (PAW) formalism [47]. Optimization of the geometry of the studied models and calculation of the thermodynamics of the water-splitting reaction were carried out using the GGA-PBE (Perdew–Burke–Ernzerhof) exchange correlation functional [48]. The on-site Coulomb correlation of *d*-electrons was taken into account by employing Hubbard corrections in the Dudarev parametrization [49] with a *U*_eff_ = *U_c_* − *J* value of 2.6 eV for titanium [50]. We must admit, on the basis of our test calculations, that the application of the *U_eff_*-parameter to Ti/Rh does not change the main conclusions/results regarding the surface free energy diagrams. Contrarily, the calculations of optical properties require accurate electronic band structures. We therefore applied the hybrid HSE06 density functional to calculate the electronic density of states and optical absorption from the DFT+U optimized charge density [51]. The optical properties were analyzed based on the transition dipole moment matrix elements:

(7) $$D_{\sigma ,ij}=e\langle \psi_{\sigma ,i}^{KS}|r|\psi_{\sigma ,j}^{KS}\rangle$$

for transitions between the initial state (σ,i) and final state (σ,j) calculated on the basis of Kohn−Sham orbitals $\psi_{\sigma ,i}^{KS}$, where σ is a spin index, i(j) labels orbitals, and *e* is an elementary charge. The transition dipole moment was used for calculating oscillator strength:

(8) $$f_{\sigma ,ij}=\frac{4\pi m\nu_{\sigma ,ij}}{3e^{2}\hbar }{\left|D_{\sigma ,ij}\right|}^{2},$$

where *m* and *ħ* are the electron mass and Planck constant, respectively, and abd ν_σ,ij_ is the frequency of transition between the *i*th and *j*th states. Using the oscillator strengths and assuming a lack of spin–orbit coupling, the absorption spectra can then be determined as follows: α(ν) = α_α_(ν) + α_β_(ν), where $\alpha_{\sigma }\left(\nu \right)=\sum_{ij}f_{\sigma ,ij}\delta \left(\nu -v_{\sigma ,ij}\right)$.

The thermodynamic corrections for the solvation effect were calculated using VASPsol [52], allowing us to consider surface wetting through the water continuum model and distinguish between dry and wet conditions. If the continuum model applied, the wet conditions were stated. The Monkhorst–Pack grid-sampling mesh used for the bulk calculations had dimensions of 2 × 2 × 2, and that for the slab calculations had dimensions of 2 × 2 × 1, with a cutoff energy value of 520 eV. The charge distribution on the ions was studied using Bader topological analysis [42]. All calculations were carried out while taking spin polarization into account, except in the case of bare undoped slabs. It has been shown that it is important to consider spin polarized electronic structures since adsorbed species have a spin moment [53].

## 5. Conclusions

The viability of the Rh-modified TiO_2_-terminated BaTiO_3_ (001) surface for developing efficient water oxidation catalysts to be used as photoanodes in PEC systems was examined using first-principles calculations. According to our results, Rh doping has a double effect on the properties of BaTiO_3_. On the one hand, doping causes the material under study to absorb sunlight in almost the entire visible range. On the other hand, the surface Rh ion acts as an excellent catalytic center, significantly lowering the overpotential values of the electrochemical reaction. It has been shown that considering the aqueous environment influences both effects.

## Figures and Tables

**Table 1:** TiO_2_-terminated surface. Calculated Bader charges q (in |*e*|) and local magnetic moments (in *μ*_B_) for the Ti (undoped surface) and Rh (doped surface) empty sites and as well as sites occupied by O, OH, and OOH.

**TiO_2_ Surface**									
Dry		Empty site (*)		OH*		O*		OOH*	
	Species	**q**	**μ**	**q**	**μ**	**q**	**μ**	**q**	**μ**
	Ti	2.15	0	2.25	0	2.10	0	2.22	0
	O1	−1.18	0	−1.15	0	−1.15	0	−1.13	0
	O2	−1.22	0	−1.24	0	−1.19	0	−1.24	0
	Adsorbant	-	-	−0.49	0	−0.74	0.53	−0.31	0.14
Wet	Ti	2.24	0	2.24	0	2.12	0	2.21	0
	O1	−1.22	0	−1.16	0	−1.19	0	−1.15	0
	O2	−1.23	0	−1.24	0	−1.22	0	−1.24	0
	Adsorbant	-	-	−0.52	0	−0.91	0.48	−0.35	0.13
**TiO_2_:Rh surface**									
Dry	Rh	1.51	1.59	1.77	0.85	1.73	1.04	1.64	0.73
	O1	−1.06	0.17	−1.04	0.11	−1.03	0.129	−1.02	0.13
	O2	−1.11	0.15	−1.20	0.03	−1.19	0.014	−1.18	0.01
	Adsorbant	-	-	−0.37	0.86	−0.33	1.04	−0.19	0.28
Wet	Rh	1.49	1.60	1.76	0.84	1.73	1.08	1.63	0.74
	O1	−1.08	0.17	−1.08	0.11	−1.05	0.14	−1.05	0.13
	O2	−1.10	0.15	−1.11	0.03	−1.20	0.019	−1.20	0.01
	Adsorbant	-	-	−0.43	0.84	−0.46	1.08	−0.23	0.29

**Table 2:** Distance (Å) between adsorbents and undoped and doped TiO_2_-terminated catalysts’ surfaces.

Surface	Adsorbant		
	O	OH	OOH
Undoped	1.655	1.836	2.055
Rh-doped	1.754	1.897	1.902

## Data Availability

Data are contained within the article.
